# The enzymatic properties of *Arabidopsis thaliana* DNA polymerase λ suggest a role in base excision repair

**DOI:** 10.1007/s11103-023-01407-8

**Published:** 2024-01-13

**Authors:** T. Morales-Ruiz, C. Beltrán-Melero, D. Ortega-Paredes, J. A. Luna-Morillo, M. I. Martínez-Macías, T. Roldán-Arjona, R. R. Ariza, D. Córdoba-Cañero

**Affiliations:** 1 Department of Genetics, University of Córdoba, Córdoba, Spain; 2 Maimónides Biomedical Research Institute of Córdoba (IMIBIC), Córdoba, Spain; 3 Reina Sofía University Hospital, Córdoba, Spain

**Keywords:** Pol λ, dRP lyase, DNA polymerase, Base excision repair, *Arabidopsis*

## Abstract

Base excision repair (BER) generates gapped DNA intermediates containing a 5′-terminal 2-deoxyribose-5-phosphate (5′-dRP) group. In mammalian cells, gap filling and dRP removal are catalyzed by Pol β, which belongs to the X family of DNA polymerases. In higher plants, the only member of the X family of DNA polymerases is Pol λ. Although it is generally believed that plant Pol λ participates in BER, there is limited experimental evidence for this hypothesis. Here we have characterized the biochemical properties of *Arabidopsis thaliana* Pol λ (AtPol λ) in a BER context, using a variety of DNA repair intermediates. We have found that AtPol λ performs gap filling inserting the correct nucleotide, and that the rate of nucleotide incorporation is higher in substrates containing a C in the template strand. Gap filling catalyzed by AtPol λ is most efficient with a phosphate at the 5′-end of the gap and is not inhibited by the presence of a 5′-dRP mimic. We also show that AtPol λ possesses an intrinsic dRP lyase activity that is reduced by mutations at two lysine residues in its 8-kDa domain, one of which is present in Pol λ exclusively and not in any Pol β homolog. Importantly, we also found that the dRP lyase activity of AtPol λ allows efficient completion of uracil repair in a reconstituted short-patch BER reaction. These results suggest that AtPol λ plays an important role in plant BER.

**Supplementary Information:**

The online version contains supplementary material available at 10.1007/s11103-023-01407-8.

## Key message

Pol λ, the only X family DNA polymerase present in plants, possesses a dRP lyase activity that is required for completion of base excision repair in *Arabidopsis*.

**Supplementary Information:**

The online version contains supplementary material available at 10.1007/s11103-023-01407-8.

## Introduction

The genome integrity of all living organisms is constantly threatened by endogenous and exogenous sources of DNA damage (Lindahl 1993). Endogenous sources include oxidation by reactive oxygen species (ROS), alkylation, mismatch of DNA bases, topoisomerase-DNA complexes, spontaneous base deamination and abasic (apurinic/apyrimidinic, AP) sites. Important exogenous sources are ionizing radiation (IR), ultraviolet (UV) radiation and exposure to chemical agents with genotoxic capacity (Chatterjee and Walker 2017). To cope with such variety of DNA insults, cells have developed a robust DNA damage response (DDR) that activates several DNA repair pathways and DNA damage checkpoints. In addition, certain types of DNA lesions are substrates for DNA damage tolerance pathways (Chatterjee and Walker 2017). The major DNA repair pathways comprise direct reversal of DNA damage, base excision repair (BER), single-strand break repair (SSBR), nucleotide excision repair (NER), mismatch repair (MMR) and double-strand break repair (DSBR) by homologous recombination (HR) or non-homologous end joining (NHEJ). Amongst such mechanisms, BER is required for the repair of a broad range of modified DNA bases (alkylated, oxidized or deaminated) and AP sites (Krokan and Bjoras 2013).

BER is initiated by DNA glycosylases that remove the damaged or modified bases generating AP sites, that are repaired either by AP endonucleases or by the AP lyase activity associated with bifunctional DNA glycosylases (Demple and Harrison 1994; Krokan and Bjoras 2013; Seeberg et al. 2000). AP endonucleases hydrolyze DNA at the 5′-side of the AP site, leaving 3′-hydroxyl (3′-OH) and 5′-deoxyribose phosphate (5′-dRP) termini (Levin and Demple 1990). AP lyases cleave 3′ to the AP site by β-elimination, generating 3′-phospho-α, β-unsaturated aldehyde (3′-PUA) and 5′-phosphate (5′-P) termini. A subset of AP lyases catalyze β-δ-elimination and generate 3′-phosphate (3′-P) termini (Levin and Demple 1990). Therefore, AP endonucleases and AP lyases generate single-nucleotide gaps with 5′- and 3′-blocked ends, respectively. Once the blocked termini have been processed to canonical 5′-P and 3′-OH ends, gap filling may proceed by insertion of either one nucleotide (short-patch BER, SP-BER) or several nucleotides (long-patch BER, LP-BER) (Cordoba-Cañero et al. 2009; Fortini and Dogliotti 2007). In mammals, DNA polymerase β is involved in gap filling during SP-BER (Srivastava et al. 1998), whereas LP-BER requires replicative DNA polymerases Pol δ and Pol ε (Levin et al. 1997). The last BER step is DNA ligation that, in mammals, is carried out by the complex of XRCC1 and LigIIIα during SP-BER (Nash et al. 1997). In LP-BER Pol δ/ε displace the strand containing the 5′-dRP terminus generating a flap structure that is processed by the flap endonuclease (FEN1), creating a nick that is sealed by LIG1 (Levin et al. 1997).

Since AP endonucleases generate a 5′-dRP blocked end, SP-BER requires a 5′-dRP lyase activity to produce 5′-P ends amenable to DNA ligation. In mammals, the mayor dRP lyase activity is intrinsic to Pol β (Srivastava et al. 1998), through an N-terminal 8-kDa domain characteristic of X family of DNA polymerases (Beard and Wilson 2000). The members of the mammalian X family of DNA polymerases are Pol β, Pol λ, Pol μ and terminal deoxyribonucleotidyl transferase (TdT), and they are mainly involved in gap filling during DNA repair (Beard and Wilson 2000). The X family proteins are distributive polymerases involved in synthesis of short segments of DNA. All these enzymes are composed of a DNA polymerization domain at the C-terminus, a DNA binding domain in the central region of the protein and, except for Pol β, a BRCT (BRCA1 C-terminal) domain at the N-terminus (Uchiyama et al. 2009). Both mammalian Pol β and Pol λ display DNA polymerase and 5′-dRP lyase activity (Garcia-Diaz et al. 2001; Matsumoto and Kim 1995), which is needed for SP-BER (Braithwaite et al. 2010). Pol λ, Pol μ and TdT, through their BRCT domain, are involved in NHEJ to repair double-strand DNA breaks generated by DNA damage, and/or V(D)J recombination to create diversity in the immunoglobulins (Fan and Wu 2004; Lee et al. 2004; Mahajan et al. 2002; Nick McElhinny et al. 2005).

Plants possess homologs of most BER genes found in other organisms, but there are exceptions. For instance, Pol β and Lig III, involved in SP-BER in mammals, are absent in plants. On the other hand, some BER proteins are only found in plants, indicating that there are some plant-specific BER features that have appeared during evolution (Roldan-Arjona et al. 2019). Since there are no plant homologs of Pol β and LigIII, it was initially believed that plants, unlike mammals, do not use SP-BER to repair damaged bases (Uchiyama et al. 2008). However, it has been reported that *Arabidopsis* cell extracts catalyze DNA repair of uracil and AP sites by both LP- and SP-BER (Cordoba-Cañero et al. 2009). However, the identity of the DNA polymerase(s) involved in plant BER is still unknown.

The only member of the X family of DNA polymerases present in higher plants is Pol λ (Uchiyama et al. 2004). By contrast, in the unicellular alga *Chlamydomonas reinhardtii*, in addition to Pol λ, sequences with similarity to X family members Pol μ and TdT have been identified (Morales-Ruiz et al. 2018)*.* The possible role of plant Pol λ in DNA repair has been studied both in rice and *Arabidopsis* (Amoroso et al. 2011; Furukawa et al. 2015; Garcia-Diaz et al. 2000; Roy et al. 2011; Uchiyama et al. 2004). In *Arabidopsis* it has been reported that, as its mammalian homolog, Pol λ performs error-free translesion synthesis past 8-oxoguanine (8-oxoG) (Amoroso et al. 2011; Maga et al. 2008). Both *Arabidopsis* and rice Pol λ have been described to have a highly conserved PCNA-interacting protein-box (PIP box) motif. In *Arabidopsis* the PIP box is involved in mediating the interaction of Pol λ with PCNA2 (proliferating cell nuclear antigen 2) to enhance the efficiency and fidelity of translesion synthesis (Amoroso et al. 2011). Moreover, it has been shown that *Arabidopsis* Pol λ plays a role in NER of UV-B induced DNA damage (Roy et al. 2011). It has been suggested that *Arabidopsis* Pol λ also participates in double-strand break repair. Specifically, an *Arabidopsis* Pol λ T-DNA insertion mutant showed sensitivity to both gamma-irradiation and treatment with radiomimetic agents, such as bleomycin, but not to others DNA damaging treatments including methyl methanesulfonate (MMS) and mitomycin-c (MMC) (Furukawa et al. 2015). All these reports suggest that Pol λ is an important component of the plant DNA damage response. However, our knowledge about the role of plant Pol λ in specific DNA repair processes, such as BER, is still very limited. The biochemical characterization of rice Pol λ indicates that it displays dRP lyase activity (Uchiyama et al. 2004). Nonetheless, although some biochemical properties of *Arabidopsis* Pol λ have been described, there is no evidence reported of its dRP lyase activity (Amoroso et al. 2011; Roy et al. 2011). In this work, we have biochemically characterized the enzymatic activity of *Arabidopsis* Pol λ and, importantly, we have found two residues, K248 and K255, within the 8-kDa domain characteristic of the X family of DNA polymerases, that are needed to repair 5′-dRP blocked ends. Our results show that *Arabidopsis* Pol λ displays both DNA polymerization and dRP lyase activities on DNA substrates mimicking DNA repair intermediates and suggest it could play an important role in plant BER.

## Material and methods

### DNA substrates

Oligonucleotides used (Supplementary Table S1) were synthesized by Integrated DNA Technologies (IDT) and purified by PAGE or dual HPLC before use. Double-stranded DNA substrates were prepared by mixing a 5 µM solution of a fluorescein (Fl) or alexa (Al) labelled oligonucleotide with a 10 µM solution of an unlabelled complementary oligonucleotide. DNA substrates with one nucleotide gap with a 3′-OH end were prepared by mixing a 5 µM solution of a 5′-alexa (Al) labelled oligonucleotide (Al_28-OH) with a 10 µM solution of both unlabelled oligonucleotide, corresponding to the complementary strand (CGR_G, CGR_A, CGR_T or CGR_C) and to the oligonucleotide containing a 5′-P, 5′-OH or 5′-THF terminus (P_30-51, OH_30-51 or THF_30-51, respectively). Annealing was carried out by heating at 95 °C for 5 min followed by slowly cooling to room temperature. DNA substrates with a 5′-dRP end were generated by incubating an oligonucleotide duplex containing a U:G mispair with 0.5 U of *Escherichia coli* uracil DNA glycosylase (UDG) (New England Biolabs) and 10 U of human AP endonuclease 1 (APE-1) (New England Biolabs, NEB).

### Protein expression and purification

The full-length *Arabidopsis thaliana* Pol λ (AtPol λ) cDNA, obtained from the *Arabidopsis* Biological Resource Center (pENTR_221-At1G10520), was subcloned into pET28a expression vector (Novagen) to add a polyhistidine (His_6_) tag at the N-terminus of AtPol λ protein. Expression was carried out in *E. coli* BL21 (DE3) *dcm*^*−*^ Codon Plus cells (Stratagene) by adding 1 mM isopropyl-1-thio-β-D-galactopyranoside. His-AtPol λ was purified by affinity chromatography on a Ni^2+^-Sepharose column (HisTrap HP; GE Healthcare). Protein was eluted with a 5 mM to 1 M gradient of imidazole and analyzed by SDS/PAGE (10%) using broad-range molecular weight standards (Bio-Rad).

### Other enzymes and reagents

*Escherichia coli* UDG and human APE1 were obtained from NEB. Human Pol β was purchased from Trevigen, T4 DNA ligase was obtained from Promega and Taq DNA Polymerase from Bioline. Anti-AtPol λ antibodies were generated by injecting rabbits with His-AtPol λ.

### Site-directed mutagenesis

Site-directed mutagenesis was performed using the Quick-Change II XL kit (Stratagene). The mutations were introduced into the expression vector pET28a (Novagen) containing the full-length wild-type (WT) *AtPol* λ cDNA using specific oligonucleotides (Supplementary Table S2). Mutational changes were confirmed by DNA sequencing and the constructs were used to transform *Escherichia coli* BL21 (DE3) *dcm*^*−*^ Codon Plus cells (Stratagene). Mutant proteins were expressed and purified as describe above.

### Gap-filling assay

Reactions (10 μl) contained 50 mM Tris HCl pH 7.5, 1 mM DTT, 0.2 mg/ml BSA, 2% glycerol, 10 mM MgCl_2_, 100 nM DNA substrate, the indicated amount of AtPol λ and dCTP, dNTPs (deoxynucleotides) or ddNTPs (dideoxynucleotides). When indicated, AtPol λ was pre-incubated with pre-immune serum or anti-AtPol λ antibody for one hour at 4 °C. After incubation at 37 °C for the indicated times, reactions were stopped by adding 20 mM EDTA, 0.6% SDS and 0.5 mg/ml proteinase K, and mixtures were incubated at 37 °C for 30 min. DNA was extracted with phenol:chloroform:isoamyl alcohol (25:24:1) and ethanol precipitated at -20 °C in the presence of 0.3 mM NaCl and 16 mg/ml glycogen. Samples were resuspended in 10 µl of 90% formamide and heated at 95 °C for 5 min. Reaction products were separated in a 12% denaturing polyacrylamide gel containing 7 M urea. Labelled DNA was visualized using FLA-5100 imager (Fujifilm) and analyzed using Multi Gauge software version 3.0 (Fujifilm).

### dRP lyase assay

Reactions (50 µl) contained 45 mM HEPES–KOH pH 7.8, 70 mM KCl, 5 mM MgCl_2,_ 1 mM DTT, 0.4 mM EDTA, 2 mM ATP, 36 µg BSA, 1 mM NAD, 0.2% glycerol, 22 mM phosphocreatine, 2.5 ng creatine kinase, 0.02 mM dCTP, and a DNA substrate with a 5′-dRP (100 nM) prepared as described above. When indicated, human Pol β (2.4 U) or AtPol λ (10 nM), were added. After incubation at 30 °C for 90 min, reaction products were stabilized by the addition of freshly prepared sodium borohydride (NaBH_4_; Sigma-Aldrich) to a final concentration of 300 mM and incubated on ice for 30 min. Subsequently, the samples were desalted using microspin G-25 columns (GE Healthcare). The reactions were stopped, DNA was extracted, and samples were processed as described above.

### In vitro reconstitution of base excision repair

BER reactions (50 μl) were performed in 45 mM HEPES–KOH pH 7.8, 70 mM KCl, 5 mM MgCl_2_, 1 mM DTT, 0.4 mM EDTA, 2 mM ATP, 36 μg BSA, 1 mM NAD, 0.2% glycerol, 22 mM phosphocreatine, 2.5 ng creatine kinase, 0.02 mM dCTP, and a DNA duplex containing a U:G mispair (100 nM). All reactions contained UDG (0.5 U) and APE1 (10 U). When indicated, human Pol β (2.4 U), AtPol λ (10 nM), and/or T4 DNA ligase (1.5 U), were added. Mixtures were incubated at 30 °C for 90 min, and the reactions were stopped, DNA was extracted, and samples were processed as described above.

## Results

### Characterization of AtPol λ gap filling activity

**Fig. 1:**
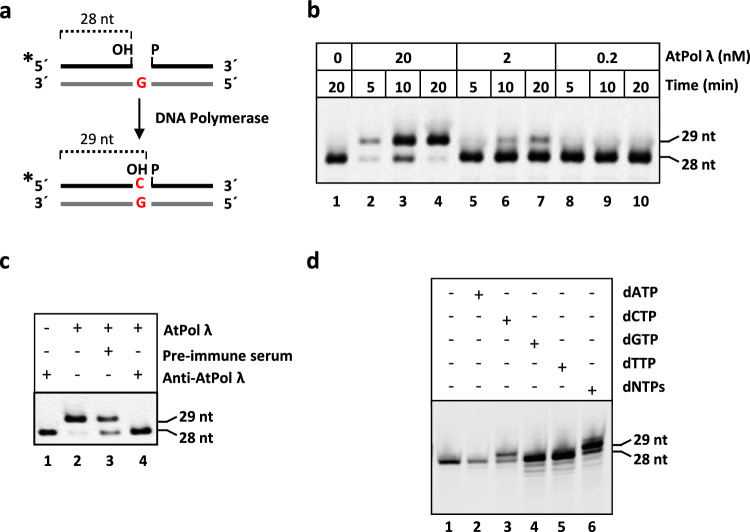
Arabidopsis Pol λ gap filling activity. **a** *Schematic diagram of substrate and product*. **b** *AtPol λ* *gap filling assay*. DNA substrates (100 nM) were incubated at 30 °C with AtPol *λ* at the concentration and time indicated in reaction mixtures containing dCTP (100 μM). **c** *Inhibition of DNA synthesis by anti-AtPol λ antiserum*. AtPol λ protein (10 nM) was pre-incubated with pre-immune serum or anti-AtPol λ antiserum for one hour at 4 °C and then incubated for 20 min at 30 °C with the DNA substrate (100 nM) in reaction mixtures containing dCTP (100 μM). **d** *DNA synthesis catalyzed by AtPol λ is template-directed*. AtPol λ protein (10 nM) was incubated with DNA substrates (100 nM) at 30 °C for 30 min in reaction mixtures containing 20 μM of dATP, dCTP, dGTP or dTTP, as indicated

In order to characterize the catalytic activity of *Arabidopsis thaliana* Pol λ, a recombinant protein (AtPol λ) was expressed in *E. coli* and purified. Subsequently, optimal conditions for detecting polymerase activity were determined by performing polymerization assays with varying enzyme concentrations. A DNA duplex with the upper strand labelled at the 5′-end and containing a single nucleotide gap flanked by 3′-OH and 5′-P ends was used as substrate (Fig. 1a). AtPol λ already showed DNA polymerase activity in a 10 min reaction at a 2 nM concentration of recombinant protein (Fig. 1b, lane 6). To confirm that the polymerase activity detected was intrinsic to AtPol λ and not due to contamination with bacterial polymerases, further polymerization assays were carried out in which AtPol λ was pre-incubated with serum generated against AtPol λ (anti-AtPol λ) (Fig. 1c). We found a complete inhibition of the polymerase gap-filling capacity in the presence of the anti-AtPol λ serum (Fig. 1c, lane 4), suggesting that the DNA polymerase activity is intrinsic to AtPol λ.

**Fig. 2:**
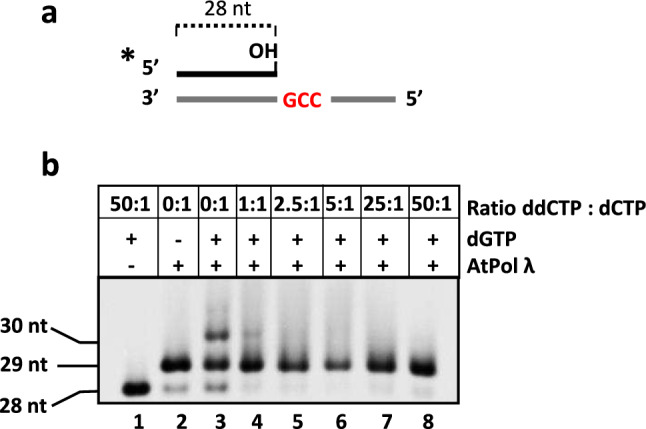
AtPol λ sensitivity to dideoxynucleotides.** a** *Schematic diagram of molecules used as DNA substrates.* **b** *DNA polymerase assay.* DNA substrate (100 nM) was incubated with AtPol λ protein (10 nM) for 60 min at 30 °C in reaction mixtures containing dCTP (10 μM), dGTP (10 μM) and increasing levels of ddCTP (0, 10, 25, 50, 250 and 500 μM) as indicated

To determine whether AtPol λ is able to insert the correct nucleotide in a gapped DNA mimicking a BER intermediate, we performed polymerization assays using a DNA substrate containing a single-nucleotide gap opposite G (Fig. 1a) in the presence of dATP, dCTP, dGTP, dTTP or a mixture of all four dNTPs (Fig. 1d). AtPol λ only incorporated a nucleotide opposite G when dCTP or dNTPs were present in the reaction (Fig. 1d, lanes 3 and 6). We also examined how the structure of the deoxyribose affects nucleotide incorporation by AtPol λ by analyzing the effect of dideoxynucleotides (ddNTPs) on its DNA polymerase activity. DNA polymerization is blocked during replication when a ddNTP is incorporated due to the lack of a canonical 3′-OH. While replicative DNA polymerases are resistant to ddNTP inhibition because they fail to bind the nucleotide analogue, human Pol β is ddNTP-sensitive because its active site can accommodate ddNTPs and incorporate them as well as dNTPs (Cavanaugh et al. 2010). Previous studies have shown that human and *O. sativa* Pol λ are also sensitive to ddNTPs and therefore unable to discriminate between dNTPs and ddNTPs (Garcia-Diaz et al. 2002; Uchiyama et al. 2004). Additionally, we have previously shown that BER catalyzed by *Arabidopsis* whole cell extracts is partially sensitive to ddNTPs (Cordoba-Cañero et al. 2009). To investigate whether AtPol λ is affected by ddNTPs, a polymerization assay was performed using a primer-template DNA substrate in which dGMP insertion requires the previous incorporation of dCMP (Fig. 2). In the absence of ddCTP, AtPol λ generated products of 29 and 30 nucleotides, corresponding to the insertion of dCMP and dGMP, respectively (Fig. 2, lane 3). However, in the presence of increasing ddCTP:dCTP ratios, a near complete inhibition of AtPol λ activity was observed (Fig. 2, lanes 4–8). A strong inhibition was already detected with a 1:1 ddCTP:dCTP ratio (Fig. 2, lane 4). Therefore, AtPol λ exhibits high sensitivity to ddNTPs, in agreement with the weak discrimination of the 3′-OH group of the nucleotide to be inserted that is displayed by the X family of DNA polymerases (Banos et al. 2008; Garcia-Diaz et al. 2002; Prasad et al. 1993).

### Effect of the template base on gap filling activity of AtPol λ

**Fig. 3:**
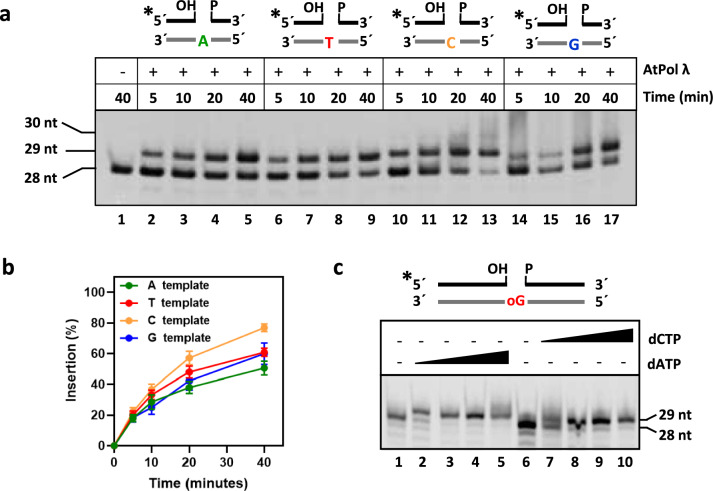
Effect of the template base on AtPol λ gap-filling activity. **a***Gap-filling assay.* DNA substrates (100 nM) containing a single-nucleotide gap with A, T, C or G as template were incubated with AtPol λ (10 nM) for 5, 10, 20 and 40 min at 30 °C in reaction mixtures containing 1 μM of the appropriate complementary deoxynucleotide. **b** *Percentage of nucleotide insertion in gaps with A, T, C or G as template*. Values are the mean with standard error from three independent experiments. **c** *Bypass of 8-oxoG by AtPol λ*. DNA substrate with a single-nucleotide gap opposite 8-oxoG (100 nM) was incubated with AtPol λ (10 nM) for 30 min at 30 °C in reaction mixtures containing increasing levels of dCTP or dATP (0, 1, 5, 10 and 50 μM) as indicated

To further analyze the DNA polymerase activity of AtPol λ, we performed a time-course DNA polymerization assay using DNA duplexes with a gap flanked by 3′-OH and 5′-P ends containing different bases (A, T, C, or G) on the complementary strand (Fig. 3a). As expected, AtPol λ performed gap-filling in all four substrates, although the efficiency of DNA synthesis was somewhat different depending on the template base (Fig. 3a-b). We found that AtPol λ displays a significant preference for C as template in comparison with A (Fig. 3b; t-test p < 0,05 and < 0,01 at 20 and 40 min, respectively). In the DNA substrate containing C as template, the presence of another C downstream in the complementary strand allowed limited strand displacement accompanied of a second dGMP insertion (Fig. 3a, lanes 12–13).

Some DNA polymerases involved in BER are capable of bypassing lesions on the template strand, resulting in both error-prone and error-free DNA synthesis (Krokan and Bjoras 2013). The ubiquitous oxidative DNA lesion 8-oxoG is highly mutagenic, as it can pair with both cytosine and adenine, leading to G:C → T:A transversions after replication (Krokan and Bjoras 2013). To analyze the translesion DNA synthesis capacity of AtPol λ, we performed a gap-filling assay with a DNA substrate containing 8-oxoG opposite the gap in the presence of either dCTP or dATP (Fig. 3c). The results indicate that AtPol λ can effectively bypass 8-oxoG by incorporating dCMP or dAMP with similar efficiencies (Fig. 3c).

### Effect of the 5′-end group on the gap-filling activity of AtPol λ

**Fig. 4:**
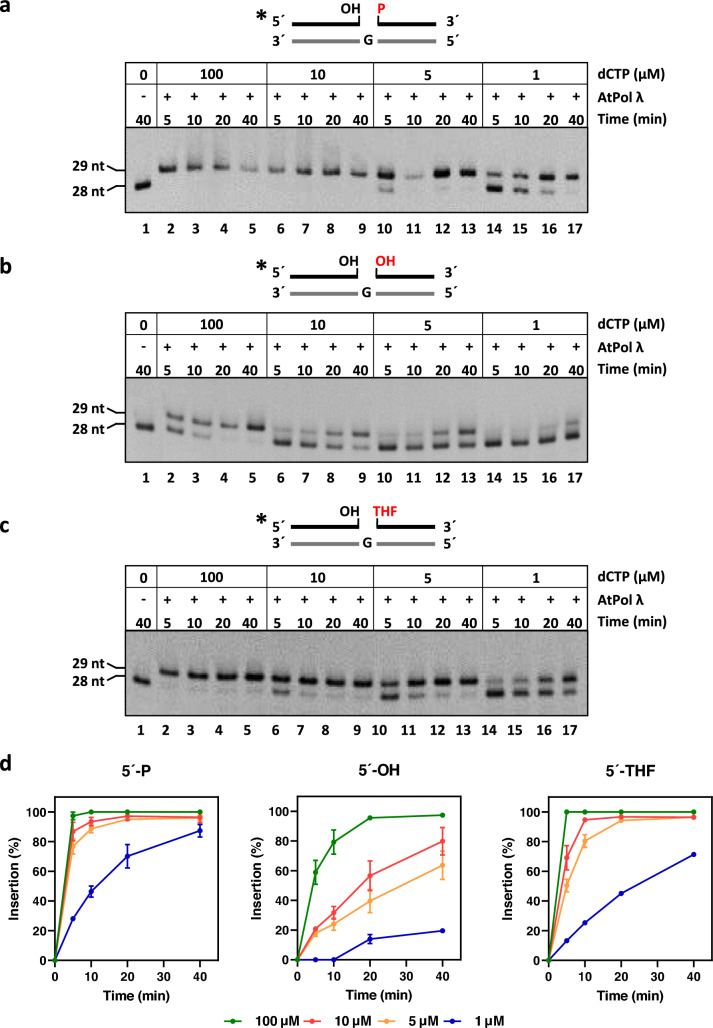
Effect the 5′-end group on AtPol λ gap-filling activity.** a–c** *Gap-filling assay.* DNA substrates (100 nM) containing a single-nucleotide gap with 5′-P (**a**), 5′-OH (**b**), or 5′-THF end (**c**) were incubated with AtPol λ (10 nM) for the indicated times at 30 °C in reaction mixtures containing dCTP at the indicated concentrations. **d** *Percentage of dCMP insertion for each DNA substrate*. Values are means ± standard errors from two independent experiments

Human Pol λ and Pol β possess high affinity for DNA substrates containing gaps flanked by 3′-OH and 5′-P ends. It has been suggested that this preference is due to the interaction between the 8-kDa domain of the protein and the phosphate group at the 5′-end of the gap (Garcia-Diaz et al. 2002; Singhal and Wilson 1993). In mammalian SP-BER initiated by AP endonucleases, the processing of the 5′-dRP group to generate a 5′-P end is a limiting step (Srivastava et al. 1998) and it has been reported that the presence of a 5′-dRP group moderately decreases the DNA polymerization activity of human Pol λ (Duym et al. 2006). To analyze the effect of the 5′-end group on the gap-filling activity of AtPol λ, we carried out DNA polymerization assays on gaps with a 5′-phosphate (5′-P), a 5′-hydroxyl (5′-OH) or a tetrahydrofuran residue (5′-THF) mimicking a 5′-dRP, which is resistant to dRP lyase activity. Time-course reactions with each substrate were performed in the presence of different dCTP concentrations (Fig. 4a–c).

When a canonical 5′-P end was used (Fig. 4a), AtPol λ incorporated a nucleotide at the shortest tested time (5 min) and at the higher dCTP concentrations of 100 and 10 μM (Fig. 4a, lanes 2 and 6), while at a lower dCTP concentrations, longer times were needed for the gap filling to occur with the same efficiency (Fig. 4a, lanes 11 and 17). However, in the presence of DNA substrates containing a 5′-OH end (Fig. 4b), AtPol λ required a longer time (20 min) for gap filling at 100 μM of dCTP (Fig. 4b, lane 4). Moreover, at lower dCTP concentrations (10, 5 and 1 μM) the percentage of product was around 80%, 60% and 20%, respectively, at the longest tested time (Fig. 4b, lanes 9, 13 and 17). When AtPol λ was incubated with the substrate containing a 5′-THF end and the lowest dCTP concentration (1 μM) the polymerase was able to fill the gap as well as with the 5′-P end (Fig. 4c, lane 17), and the minor differences were not statistically significant. At higher dCTP concentrations (100, 10 and 5 μM), the polymerase performed gap filling generating a product of 29 nt in 5, 10 and 20 min, respectively (Fig. 4c, lanes 2, 7 and 12). Altogether, these results suggest that the gap-filling capacity of AtPol λ is highest on a gap with a 5′-P end, and strongly inhibited by a 5′-OH end (Fig. 4d). Importantly, the presence of a THF group at the 5′-side of the gap did not have any inhibitory effect on the enzyme efficiency, suggesting that AtPol λ can effectively fill DNA repair gaps with a 5′-dRP.

### AtPol λ possesses an intrinsic dRP lyase activity

A limiting step of SP-BER is the removal of the 5′-dRP generated after the processing of abasic sites by AP endonucleases. In humans, the removal of this end is mainly carried out by the dRP lyase activity of polymerase β (Beard and Wilson 2000). No homologs of Pol β have been described in plants and AtPol λ is the only member of the X family described so far (Uchiyama et al. 2009), suggesting that this polymerase might play a role in SP-BER. The 8-kDa domain present in all members of this family is responsible for the dRP lyase activity, which is catalyzed through β-elimination via a Schiff base intermediate between a nucleophile lysine residue and DNA (Matsumoto and Kim 1995). Specific lysine residues responsible for this activity have been identified in human Pol β (K72) (Deterding et al. 2000) and Pol λ (K312) (Garcia-Diaz et al. 2001).

**Fig. 5:**
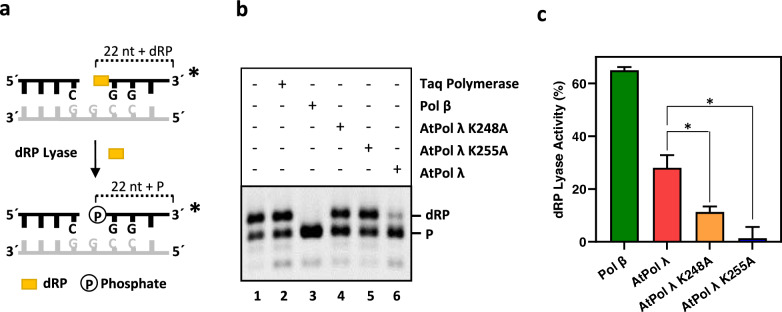
AtPol λ dRP lyase activity. **a** *Schematic diagram of substrate and product*. **b** *dRP lyase assay.* DNA (100 nM) was incubated at 30 °C for 90 min in reaction mixtures containing the indicated DNA polymerase: Taq (1 U); human Pol β (2.4 U); WT or mutant versions of AtPol λ (10 nM). **c** *Percentage of 5′-dRP lyase activity*. The percentage of 5′-dRP lyase activity was calculated as the percentage of 5′-P product generated by each sample subtracting the 5′-P product generated by spontaneous hydrolysis of the 5′-dRP end in the absence of enzyme. Values are means ± standard errors from three independent experiments. Asterisks indicate statistically significant differences (P < 0.05; Student’s t-test)

In order to test whether AtPol λ has intrinsic dRP lyase activity, we first identified candidate nucleophile residues in its 8-kDa domain. By multiple sequence alignment we found that AtPol λ has a histidine (H260) at the orthologous position of human Pol β K72 and Pol λ K312 (Supplementary Fig. S1). We therefore focused on two AtPol λ lysine residues located nearby. One of such residues (K255) is conserved in all Pol λ orthologs, but not in Pol β enzymes, whereas the other one (K248) is only conserved in plant Pol λ proteins (Supplementary Fig. S1). To examine the role of K248 and K255, two mutant derivatives (AtPol λ K248A and AtPol λ K255A) were expressed and purified as recombinant His tagged proteins. Subsequently, dRP lyase activity assays were performed using AtPol λ and its mutant versions (Fig. 5a). The removal of the 5′-dRP group generates a fragment with a 5′-P end that exhibits greater electrophoretic mobility. Although the non-processed 5′dRP end was stabilized by treatment with borohydride, spontaneous appearance of 5′-P was observed in the absence of any enzyme (Fig. 5b, lane 1) and in the presence of a DNA polymerase without dRP lyase activity (Fig. 5b, lane 2), suggesting that some spontaneous 5′-dRP hydrolysis takes place during the incubation time. We found that AtPol λ exhibited a clearly detectable dRP lyase activity (Fig. 5b, lane 6) that was significantly reduced in the mutant versions K248A and, particularly, K255A (Fig. 5b-c). These results indicate that AtPol λ possesses an intrinsic dRP lyase activity and suggest that both K248 and K255 are involved in this enzymatic function.

**Fig. 6:**
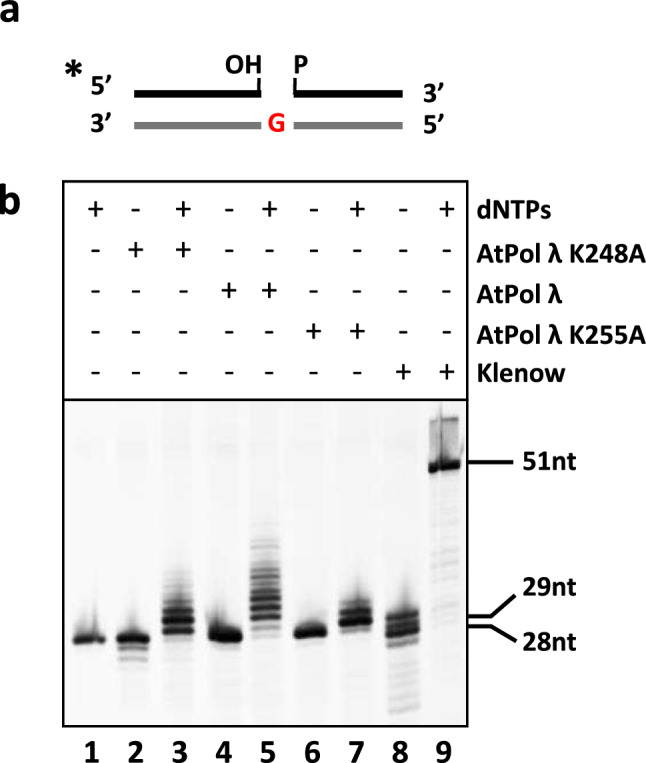
DNA polymerase activity of AtPol λ K248A and K255A mutant proteins. **a** *Schematic diagram of the DNA substrate*. **b** *DNA polymerase assay.* DNA (100 nM) was incubated for 30 min at 30 °C with AtPol λ WT or mutant versions (10 nM) in reaction mixtures with or without dNTPs (100 μM), as indicated. Klenow DNA polymerase (2.5 U) was used as a control

Next, we asked whether mutations in K248 or K255 have any effect on the DNA polymerase activity of AtPol λ. For this purpose, a polymerization assay was carried out using a DNA substrate containing a gap flanked by 3′-OH and 5′-P ends (Fig. 6). As compared with Klenow DNA polymerase, which achieved full-length DNA synthesis by displacement of the top strand (Fig. 6, lane 9), AtPol λ exhibited a limited capacity for strand displacement inserting only between 8 and 10 nucleotides (Fig. 6, lane 5). AtPol λ did not exhibited 3′-5′ exonuclease activity when the incubations were carried out in the absence of dNTPs (Fig. 6, lane 4). In comparison, mutant AtPol λ K248 showed a DNA polymerization activity similar to that of the WT protein, but a significantly lower capacity for strand displacement (Fig. 6, lane 3). Also, it exhibited a detectable 3′-5′ exonuclease activity in the absence of dNTPs (Fig. 6, lane 2). The K255 mutant version of AtPol λ displayed a stronger reduction in strand displacement capacity (Fig. 6, lane 7), but did not show detectable 3′-5′ exonuclease activity (Fig. 6, lane 6). These results suggest that residues K248 and K255 are not required for DNA polymerase activity but modulate the capacity of AtPol λ to perform strand displacement during gap-filling.

### AtPol λ 5′-dRP lyase activity is required for efficient completion of SP-BER in vitro

**Fig. 7:**
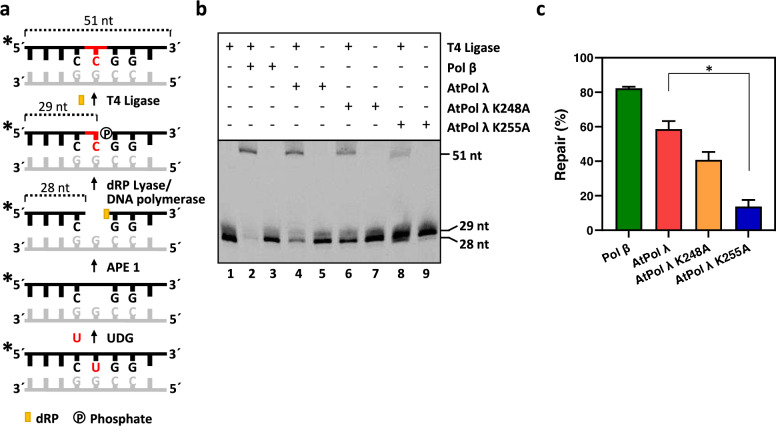
AtPol λ dRP lyase activity is required for SP-BER in vitro. **a** *Schematic diagram of the BER reconstitution assay*. **b** *BER assay.* DNA (100 nM) was incubated at 30 °C for 90 min in reaction mixtures containing 20 μM dCTP, *E. coli* UDG (0.5 U), human APE1 (10 U) and, when indicated, T4 DNA Ligase (1.5 U), human Pol β (2.4 U), or AtPol λ (10 nM). **c** *Percentage of fully repaired DNA products*. Values are means ± standard errors from two independent experiments. Asterisks indicate statistically significant differences (P < 0.05; Student’s t-test)

The dRP lyase activity of human Pol β, which removes the blocking dRP group at the 5′-end of the gap, is required to allow repair completion during SP-BER (Srivastava et al. 1998). To determine whether AtPol λ also carries out this function, an in vitro SP-BER reconstitution assay was performed using recombinant proteins and a DNA substrate containing a U:G mispair (Fig. 7). In this assay, uracil is removed by UDG leaving an intact AP site that is cleaved by APE1 at the 5′-side generating a gap flanked by 3′-OH and 5′-dRP ends. After nucleotide insertion by a DNA polymerase, a dRP lyase activity is required before DNA ligation completes repair (Fig. 7a). The results show that reactions containing both DNA ligase and AtPol λ, but not those with DNA ligase alone, generated a fully repaired product detected as a 51-nucleotide fragment (Fig. 7b, lanes 1 and 4). Although lower, repair levels achieved with AtPol λ were close to those observed with Pol β (Fig. 7c). Importantly, we found that the levels of fully repaired products were significantly lower in reactions containing the AtPol λ K255A mutant protein (Fig. 7c). Taken together, our results suggest that AtPol λ can support SP-BER, and that its dRP lyase activity is required for efficient completion of repair.

## Discussion

In this work we have performed a biochemical characterization of *A. thaliana* DNA polymerase λ and explored its possible role in BER. Firstly, we have shown that, as the rice and human orthologs (Garcia-Diaz et al. 2002; Uchiyama et al. 2004), AtPol λ inserts the correct nucleotide in a single-nucleotide gap. We have also shown that AtPol λ is able to catalyze DNA synthesis at the lowest dNTP concentration tested (1 μM), which is consistent with the high affinity for dNTPs observed in human Pol λ (Garcia-Diaz et al. 2002). We also showed that AtPol λ is inhibited by ddCTP, even at low ddCTP: dCTP ratios. This result suggests a low discrimination for the presence of the 3′-OH group in the sugar of the incoming deoxynucleotide, as it has been found in other X family DNA polymerases (Banos et al. 2008; Garcia-Diaz et al. 2002; Prasad et al. 1993). We have found that, like human Pol β and Pol λ, AtPol λ does not possess an intrinsic 3′-5′ exonuclease activity that might remove a nucleotide with the wrong sugar. The ddNTP resistance of high-fidelity DNA polymerases is not due to their 3′-5′-exonuclease activity, but to an exquisite structural selectivity that prevents productive binding to deoxynucleotides lacking hydroxyl at C3′ (Wang et al. 2012). Such selectivity is absent in ddNTP-sensitive DNA polymerases. For Pol β and other members of the X family, it has been proposed that a conserved Arg residue (R183 in human Pol β and R420 in human Pol λ) is sufficient to stabilize the incoming nucleotide in the absence of O3′ (Cavanaugh et al. 2010). Interestingly, this residue is also conserved in AtPol λ (R370), which suggests that the structural basis for low deoxynucleotide discrimination is conserved across Pol β and Pol λ proteins.

Interestingly, we found an effect of the base opposite the gap on the polymerization efficiency of AtPol λ. The rate of nucleotide incorporation catalyzed by the enzyme was higher in substrates containing a C in the template strand, as compared to G, T or A. Such preference might be related to the type of initial DNA damage that leads the generation of the repair gap. A cytosine opposite the gap is found in DNA intermediates arising during repair of 8-oxoG. This suggests that AtPol λ could participate, preferably but not exclusively, in the repair of certain DNA lesions. In human cells, Pol β has been implicated in 8-oxoG BER initiated by OGG1 DNA glycosylase (Dianov et al. 1998; Fortini et al. 1999). Plants possess orthologs of OGG1 and FPG, both of which are involved in 8-oxoG repair (Cordoba-Cañero et al. 2014). Therefore, it will be interesting to analyse whether AtPol λ functions in DNA repair of 8-oxoG initiated by OGG1 and/or FPG.

Besides DNA repair functions, Pol λ enzymes are thought to play a role in DNA damage tolerance through DNA translesion synthesis. Thus, mammalian and *Arabidopsis* Pol λ have been reported to perform both error-free and error-prone bypass of 8-oxoG lesions (Amoroso et al. 2011; Picher and Blanco 2007). Here, we have confirmed that AtPol λ can catalyze both the correct incorporation of C and misincorporation of A opposite 8-oxoG. In our analysis, we have used a one-nucleotide gap mimicking a DNA intermediate generated during BER of 8-oxoG:A mispairs. In human cells such mispairs are targets of MUTYH DNA glycosylase, which specifically excises the misincorporated A and leaves 8-oxoG in the repair-template strand (Slupska et al. 1996). Since *Arabidopsis* possesses a MUTYH ortholog (Roldan-Arjona et al. 2019), the capacity of AtPol λ to misincorporate A opposite 8-oxoG must be somehow reduced in vivo in order to avoid futile cycles of 8-oxoG:A repair. In fact, it has been reported that PCNA2 increases AtPol λ fidelity in translesion DNA synthesis (Amoroso et al. 2011).

It has been previously shown that human Pol λ is a distributive DNA polymerase on a template-primer substrate but processive in short gaps containing a phosphate group at its 5′-end (Garcia-Diaz et al. 2002). This is believed to be a consequence of the additional contacts that are made between the N-terminal 8-kDa domain of the protein and the 5′-end of the downstream strand in the gapped substrate (Garcia-Diaz et al. 2004). Here we have shown that AtPol λ is able to perform gap-filling in the presence of different groups at the 5′-end of the gap but shows the highest polymerization efficiency when the 5′-end contains a phosphate group. This finding suggests that plant Pol λ is not specialized in DNA substrates with a canonical 5′-P end and might fulfils diverse functions by being able to process substrates with different 5′-termini. We found a much-reduced, but still significant, gap-filling activity in gapped substrates containing an OH group at the 5′-terminus of the downstream strand. Such 5′-OH blocked ends are found at DNA breaks induced by abortive activity of topoisomerase I (TOP1) which are generated by TOP1 inhibitors, reactive oxygen species, and other genotoxins (Caldecott 2008; Pommier et al. 2003). TOP1 cleavage complexes are repaired by tyrosyl-DNA phosphodiesterase 1 (TDP1), that removes topoisomerase I from the 3′-termini at the gap, generating a gap with 3′-P and 5′-OH ends that are converted to 3′-OH and 5′-P by the DNA phosphatase and kinase activities of PNKP, respectively (Caldecott 2008; Jilani et al. 1999; Pouliot et al. 2001). In mammals, the repair is completed in most cases by short patch gap filling mediated by Pol β (Krokan and Bjoras 2013; Kubota et al. 1996; Srivastava et al. 1998). The *Arabidopsis* ortholog of PNKP lacks kinase activity (Martinez-Macias et al. 2012; Petrucco et al. 2002), and the mechanism responsible for processing 5′-OH ends in gapped DNA repair intermediates in plants remains unidentified. It is also unknown if AtPol λ plays a role in the processing of TOP1 cleavage complexes. Interestingly, we found that the nucleotide insertion efficiency of AtPol λ when the gap contains a 5′-dRP-mimicking group (THF), is comparable to that observed with a 5′-P end. BER DNA intermediates frequently harbour 5′-dRP blocking termini that arise upon cleavage of abasic sites by APE1 (Krokan and Bjoras 2013; Levin and Demple 1990) and are processed by the dRP lyase activity of Pol β (Matsumoto and Kim 1995; Srivastava et al. 1998). However, it has been reported that the dRP lyase activity of Pol β is rate-limiting during BER, and that its DNA polymerase activity performs gap-filling prior to removal of the dRP group (Srivastava et al. 1998). Our results suggest that AtPol λ may perform efficient DNA synthesis in gapped DNA repair intermediates before elimination of the 5′-dRP moiety.

Like Pol β, human Pol λ possesses an 8-kDa domain responsible for dRP lyase activity (Garcia-Diaz et al. 2000). The human Pol λ K312 residue, which is structurally homologous to Pol β K72, is crucial for this enzymatic function, suggesting that this is the main nucleophile responsible for the reaction (Garcia-Diaz et al. 2001). Interestingly, our multiple sequence analysis revealed that human Pol λ K312 and Pol β K72 are replaced by histidine in plant Pol λ proteins. In our search for alternative candidate residues, we have found that AtPol λ K248 and K255 are important for the dRP lyase reaction. Replacement of these lysine residues by alanine caused a significant reduction of the dRP lyase activity of AtPol λ, which was nearly abolished in the K255A mutant protein. Alanine substitution for K248 resulted in a lesser, but still important reduction of activity. These results suggest that K255 is the preferred but no required nucleophile responsible for the dRP lyase reaction catalyzed by AtPol λ. Interestingly, AtPol λ K255 is conserved in plant and animal Pol λ enzymes, but not in Pol β proteins, whereas AtPol λ K248 is conserved in plant Pol λ and in Pol β proteins, but not in metazoan Pol λ. The residue aligned with AtPol λ K255 in human Pol β is K60, and its replacement by alanine also results in a significant reduction in Pol β dRP lyase activity (Prasad et al. 1998).

We found that the DNA polymerase activity of AtPol λ K248A and K255A mutant proteins was not affected, but their ability to extend DNA synthesis in a single-nucleotide gap, with the consequent displacement of the downstream strand, was impaired. This result agrees with the fact that residues in the 8-kDa domain of human Pol λ make specific contacts with the 5′-end of the downstream strand in gapped substrates (Garcia-Diaz et al. 2004). The strand-displacement ability of AtPol λ might allow its participation in LP-BER. Although this has already been suggested for human Pol λ by some authors (Garcia-Diaz et al. 2001), to date there is no evidence that AtPol λ is involved in plant LP-BER.

In this work we have also shown that the dRP lyase activity of AtPol λ is required to complete SP-BER of uracil in a reconstituted repair reaction. In the BER reconstitution assay, AtPol λ was able to perform both the gap-filling and dRP removal steps that allow the final DNA ligation of the processed strand. In contrast, the ability of AtPol λ K248A and K255A mutant proteins to promote the repair of the BER intermediate was significantly impaired. Importantly, the capacity of the mutant AtPol λ versions to support SP-BER was inversely correlated with their dRP lyase activity and was strongly reduced in the AtPol λ K255A protein. In mammalian cells, removal of the dRP group is a critical BER step in vivo, since only the dRP lyase activity of Pol β, and not its DNA polymerization capacity, is required to reverse the DNA damage sensitivity of Pol β-null cells (Sobol et al. 2000). Since plants lack Pol β homologues, our results suggest that the dRP lyase activity of AtPol λ might play a role in *Arabidopsis* SP-BER in vivo.

### Supplementary Information

Below is the link to the electronic supplementary material.

Supplementary file1 (PDF 1763 KB)

## Data Availability

The authors confirm that the data supporting the findings of this study are available within the article and its supplementary materials.
