# Supplementary material for: The enzymatic properties of Arabidopsis thaliana DNA polymerase λ suggest a role in base excision repair
Source: Plant Mol Biol. 2024 Jan 13;114(1):3. doi: 10.1007/s11103-023-01407-8 (PMC10787897; doi:10.1007/s11103-023-01407-8)
Supplement: Supplementary file 1 — Supplementary file1 (PDF 1763 KB) [file 11103_2023_1407_MOESM1_ESM.pdf]

Supplementary Information

Journal: Plant Molecular Biology

The enzymatic properties of *Arabidopsis thaliana* DNA polymerase λ suggest a role in base excision repair

Morales-Ruiz, T.[1, 2, 3]; Beltrán-Melero, C.[1, 2, 3]; Ortega-Paredes, D.[1, 2, 3]; Luna-Morillo, J. A.[1, 2, 3]; Martínez-Macías, M. I.[1, 2, 3]; Roldán-Arjona, T.[1, 2, 3]; Ariza, R. R.[1, 2, 3] and Córdoba-Cañero, D.[1, 2, 3]*

[1] Department of Genetics, University of Córdoba, Córdoba, Spain; [2] Maimónides Biomedical Research Institute of Córdoba (IMIBIC), Córdoba, Spain; [3] Reina Sofía University Hospital, Córdoba, Spain.

*Correspondence: b72cocad@uco.es; ORCID: 0000-0002-9659-4928

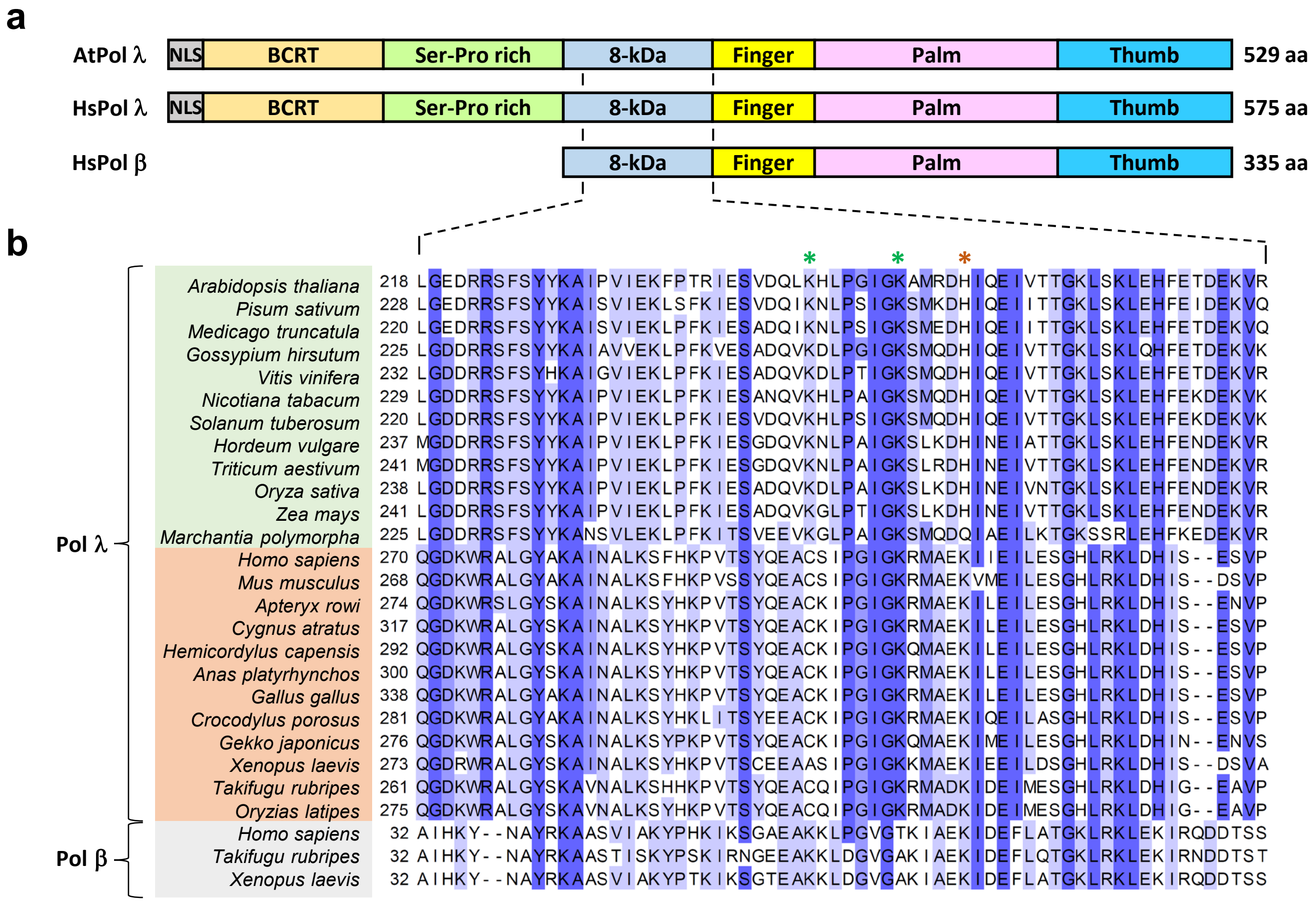

Supplementary Fig. S1 Conserved residues at the 8-kDa domain of Pol λ orthologs. a *Domain organization of Pol λ and Pol β proteins.* **b** *Multiple sequence alignment of residues from the 8-kDa domain in Pol λ and Pol β orthologs.* Green asterisks indicate positions of K248 and K255 in *Arabidopsis thaliana* Pol λ. The brown asterisk indicates the position of K312 in *Homo sapiens* Pol λ. Accession numbers (top to bottom) are: NP_172522.2 (*Arabidopsis thaliana*), XP_050905595.1 (*Pisum sativum*), XP_013448290.1 (*Medicago truncatula*), XP_016683668.2 (*Gossypium hirsutum*), XP_002266446.2 (*Vitis vinifera*), XP_016498805.1 (*Nicotiana tabacum*), XP_006355938.1 (*Solanum tuberosum*), XP_044955974.1 (*Hordeum vulgare*), KAF7107769.1 (*Triticum aestivum*), XP_015643818.1 (*Oryza sativa*), AQL00879.1 (*Zea mays*), OAE34569.1 (*Marchantia polymorpha*), NP_001167555.1 (*Homo sapiens*), NP_001317435.1 (*Mus musculus*), XP_025925320.1 (*Apteryx rowi*), XP_050567836.1 (*Cygnus atratus*), XP_053167172.1 (*Hemicordylus capensis*), XP_012958539.2 (*Anas platyrhynchos*), XP_040531078.1 (*Gallus gallus*), XP_019411772.1 (*Crocodylus porosus*), XP_015271424.1 (*Gekko japonicus*), XP_018080738.1 (*Xenopus laevis*), XP_003972614.2 (*Takifugu rubripes*), XP_004066233.1 (*Oryzias latipes*), P06746 (*Homo sapiens*), H2UYD9 (*Takifugu rubripes*) and O57383 (*Xenopus laevis*)

Table S1 Oligonucleotides used as substrates

Name[a]	DNA Sequence (5´-3´)	Strand[b]
5´Al-28OH	TCACGGGATCAATGTGTTCTTTCAGCTC	U
P_30-51	GGTCACGCTGACCAGGAATACC	U
OH_30-51	GGTCACGCTGACCAGGAATACC	U
THF_30-51	GGTCACGCTGACCAGGAATACC	U
5´Fl -UGF	TCACGGGATCAATGTGTTCTTTCAGCTCUGGTCACGCTGACCAGGAATACC	U
UGF-3´Fl	TCACGGGATCAATGTGTTCTTTCAGCTCUGGTCACGCTGACCAGGAATACC	U
CGR-oxoG	GGTATTCCTGGTCAGCGTGACCOGAGCTGAAAGAACACATTGATCCCGTGA	L
CGR-G	GGTATTCCTGGTCAGCGTGACCGGAGCTGAAAGAACACATTGATCCCGTGA	L
CGR-C	GGTATTCCTGGTCAGCGTGACCCGAGCTGAAAGAACACATTGATCCCGTGA	L
CGR-A	GGTATTCCTGGTCAGCGTGACCAGAGCTGAAAGAACACATTGATCCCGTGA	L
CGR-T	GGTATTCCTGGTCAGCGTGACCTGAGCTGAAAGAACACATTGATCCCGTGA	L

[a]Al, Alexa Fluor; P, phosphate; THF, tetrahydrofuran; Fl, Fluorescein; O, 8-oxoguanine [b]U, upper, L, lower.

Table S2 Oligonucleotides used for site-directed mutagenesis

Name	DNA Sequence[a] (5´- 3´)
K248A_F	GTTGATCAGCTCGCACACCTCCCTGGAATCGG
K248A_R	CCGATTCCAGGGAGGTGTGCGAGCTGATCAAC
K255A_F	TCCCTGGAATCGGAGCGGCAATGAGAGATCATATTCAAG
K255A_R	TTGAATATGATCTCTCATTGCCGCTCCGATTCCAGGGA

[a] Mutagenized codons are underlined.
